# Do Sleep Disturbances and Psychotic-Like Experiences in Adolescence Share Genetic and Environmental Influences?

**DOI:** 10.1037/abn0000057

**Published:** 2015-05-04

**Authors:** Mark J. Taylor, Alice M. Gregory, Daniel Freeman, Angelica Ronald

**Affiliations:** 1 Genes Environment Lifespan Laboratory, Centre for Brain and Cognitive Development, Birkbeck, University of London; 2 Department of Psychology, Goldsmiths, University of London; 3 Department of Psychiatry, University of Oxford; 4 Genes Environment Lifespan Laboratory, Centre for Brain and Cognitive Development, Birkbeck, University of London

**Keywords:** sleep, psychotic-like experiences, twin study, adolescence

## Abstract

Sleep disturbances regularly co-occur with clinical psychotic disorders and dimensions of psychotic-like experiences (PLEs). One possible explanation for this, which has yet to be tested, is that similar genetic or environmental influences underlie sleep disturbances and vulnerability to PLEs. We conducted a twin study to test this possibility in relation to sleep disturbances and six specific PLEs in adolescence in the general population. Approximately 5,000 16-year-old twin pairs completed the Pittsburgh Sleep Quality Index and Insomnia Severity Index. PLEs were assessed using the Specific PLEs Questionnaire, comprising five self-report subscales (Paranoia, Hallucinations, Cognitive Disorganization, Grandiosity, and Anhedonia) and one parent-report subscale (Negative Symptoms). The associations between these measures were tested using structural equation twin model fitting. Paranoia, Hallucinations, and Cognitive Disorganization displayed moderate and significant correlations with both sleep measures (0.32–.42), while Negative Symptoms, Anhedonia, and Grandiosity showed lower correlations (0.01–0.17). Genetic and environmental influences significantly overlapped across PLEs (Paranoia, Hallucinations, Cognitive Disorganization) and both types of sleep disturbance (mean genetic and nonshared environmental correlations = 0.54 and 0.24, respectively). These estimates reduced, yet remained significant, after controlling for negative affect. The association between PLEs with sleep disturbances in adolescence is partly due to genetic and environmental influences that are common to them both. These findings indicate that the known neurobiology of sleep disturbance may provide clues regarding the causes of PLEs in adolescence.

PLEs can be seen as milder manifestations of psychotic symptoms (Laursen, Munk-Olsen, Nordentoft, & Bo Mortensen, 2007; Dominguez, Wichers, Lieb, Wittchen, & van Os, 2011). They typically emerge during adolescence (Poulton et al., 2000; Ronald et al., 2014), prior to the typical age of onset for psychotic disorders (Laursen et al., 2007), and are thought to confer an increased risk of subsequently developing psychotic disorders (Kelleher & Cannon, 2011). Sleep disturbances, including insomnia (Freeman, Pugh, Vorontsova, & Southgate, 2009) and poor sleep quality (Chouinard, Poulin, Stip, & Godbout, 2004), are common features of psychotic disorders. Of note, these disturbances often precede the onset of psychotic disorders, occurring at elevated rates alongside PLEs. For instance, symptoms of insomnia are associated with elevated positive symptoms (Lee, Cho, Cho, Jang, & Kim, 2012), negative symptoms (Lunsford-Avery et al., 2013), and cognitive distortions (Lee et al., 2012). Such disturbances also appear to be associated with an increased chance of transitioning from an ultrahigh risk state to a first psychotic episode (Ruhrmann et al., 2010). It is thus of interest and importance to understand the association between PLEs and sleep disturbances in adolescent community-based samples.

A causal relationship between sleep disturbances and PLEs has often been postulated (Freeman et al., 2010; Freeman et al., 2012), yet ostensible overlap in the neurobiology of these phenotypes, such as the involvement of serotonin (Aghajanian & Marek, 2000; Pritchett et al., 2012), opens up the possibility that PLEs and sleep disturbances have shared genetic and environmental causes (Caron & Rutter, 1991; Neale & Kendler, 1995). Twin studies have implicated both genetic and environmental factors in sleep (Barclay & Gregory, 2013) and PLEs (Ericson, Tuvblad, Raine, Young-Wolff, & Baker, 2011; Hay et al., 2001; Hur, Cherny & Sham, 2012; Kendler et al., 2011; Lin et al., 2007; Zavos et al., 2014). A recent study further suggested that the heritability of PLEs changed across different factors, with a heritability estimate as low as 15% reported for hallucinations in males, which increased to 59% for negative symptoms (Zavos et al., 2014). The degree to which the causes of PLEs and sleep disturbances overlap in adolescence, a period during which PLEs are common in the general population (Ronald et al., 2014), is, however, unknown.

We thus aimed to investigate the degree to which genetic and environmental influences on sleep disturbances were also associated with PLEs using the classical twin design. We focused on an adolescent sample, since adolescence is a period where PLEs are relatively common in the general population (Ronald et al., 2014), yet psychotic disorders are unlikely to have yet had their onset (Kelleher & Cannon, 2011). Factor analytic studies indicate that PLEs do not represent a unitary construct; rather, they comprise a multitude of factors. Consequently, we tested genetic and environmental associations between sleep disturbances and *six specific* PLEs, including three positive symptoms (paranoia, hallucinations, and grandiosity), two negative symptoms (anhedonia and parent-rated negative symptoms), and cognitive disorganization.

Furthermore, sleep disturbances and PLEs are closely tied with negative affective states, including depression and anxiety (Buysse et al., 2007); indeed, one study suggested that the direct regression coefficient of paranoia on insomnia reduced from 0.68 to 0.25 in a mediation model that included negative affect (Freeman et al., 2010). Consequently, there is a need to study the causal relationship between PLEs and sleep disturbances in the context of negative affect; for instance, does the estimated degree of genetic and environmental overlap between these traits reduce when one takes account of negative affect?

While there have been no twin studies of the association between PLEs and sleep disturbances, evidence that sleep disturbances are linked with positive, negative, and cognitive PLEs led us to hypothesize that there would be significant associations, including causal overlap, between sleep disturbances and PLEs. Further, we also hypothesized that these associations would reduce when controlling for negative affect.

## Method

### Participants

Families of 16-year-old twins participating in the Twins Early Development Study (TEDS) were invited to take part in the Longitudinal Experiences And Perceptions (LEAP) project. TEDS is a community-based, population-representative study of twins born in England and Wales between 1994 and 1996 (Haworth, Davis & Plomin, 2013). Of the 10,874 families invited to participate in LEAP, 5,059 parents (47%) returned questionnaires and 5,076 twin pairs (47%) returned data. The participating sample was reasonably representative of the main sample (full information on the participating and nonparticipating families can be found in Table 1). Exclusions were conducted for genetic syndromes (including autism spectrum conditions, Fragile X syndrome, and cystic fibrosis), chromosomal abnormalities (including Down syndrome and cerebral palsy), extreme perinatal or prenatal complications, and missing first contact or zygosity data. The final sample comprised 4,800 pairs of twins, including 709 male monozygotic (MZ) pairs, 1,013 female MZ pairs, 664 male dizygotic (DZ) pairs, 889 female DZ pairs, and 1,525 opposite sex DZ pairs. Zygosity was ascertained through a combination of DNA testing and parent report of twin resemblance (Price et al., 2000).[Table-anchor tbl1]

### Materials

#### Specific psychotic-like experiences

The Specific Psychotic Experiences Questionnaire (SPEQ; Ronald et al., 2014) was used to assess PLEs. The measure comprised six subscales: Paranoia (15 items), Hallucinations (9 items), Cognitive Disorganization (11 items), Grandiosity (8 items), Anhedonia (10 items), and Negative Symptoms (10 items). The items were adapted from adult measures of these traits. Where necessary, the wording of the questions was adapted for age appropriateness according to expert opinions from clinicians specializing in adolescent psychosis. All subscales were self-report, except for Negative Symptoms, which was completed by parents of the twins. For Paranoia, twins were asked how frequently they had certain thoughts (e.g., “People might be conspiring against me”). For Hallucinations, twins were asked to rate how frequently they had experienced any sensory anomalies (e.g., “Hear noises or sounds when there is nothing about to explain them”). For the remaining three self-report subscales, twins were asked about these experiences during the previous month (e.g., “Do you often have difficulties in controlling your thoughts?” [Cognitive Disorganization], “I am much more unique than anyone else” [Grandiosity], “I don’t look forward to things like eating out at restaurants” [Anhedonia]). To assess Negative Symptoms, parents were asked how true a number of statements were in relation to their twins (e.g., “Often fails to smile or laugh at things others would find funny”). A full list of all the SPEQ items is provided by Ronald et al. (2014).

All six SPEQ subscales were reliable (see Table 1 for interval consistency values), and stable across a 9-month period (*r* = .65–0.74). Construct validity was established in a number of ways. Principal components analysis, reported elsewhere (Ronald et al., 2014), supported dividing the SPEQ into the six subscales outlined above. Furthermore, individuals reporting having “definitely” experienced any psychotic symptoms on an alternative measure, the psychosis-like symptoms questionnaire (PLIKS; Zammit, Owen, Evans, Heron, & Lewis (2011)) scored significantly higher on all SPEQ subscales, except for Anhedonia, relative to those who did not report any psychotic symptoms (all *p* < .001). Continuous PLIKS scores also displayed significant (*p* < .001) correlations with scores on Hallucinations (*r* = .60), Paranoia (*r* = .48), Cognitive Disorganization (*r* = .41), and Grandiosity (*r* = .27) (Ronald et al., 2014). Individuals with relatives with first- or second-degree relatives with schizophrenia and/or bipolar disorder scored higher on all SPEQ subscales, except for Anhedonia, than those who did not (all *p* < .05, except for Hallucinations).

#### Sleep disturbances

Sleep quality was assessed via a self-report measure, the Pittsburgh Sleep Quality Index (PSQI; Buysse, Reynolds, Monk, Berman, & Kupfer, 1989), a 17-item questionnaire that quantitatively assesses sleep quality. Items inquired about various aspects of sleep during the previous month, such as how long the participant typically slept each night, amount of time spent in bed each night, daytime disruption, sleep medication use, and whether sleep has been disrupted by factors such as struggling to fall and stay asleep. Scores ranged from 0–21, with higher scores indicative of poorer sleep quality. The PSQI displayed good internal consistency (see Table 1). The PSQI has good sensitivity and specificity in detecting individuals with persistent poor sleep quality (89.6% sensitivity and 86.5% specificity; Buysse et al., 1989), as well as detecting individuals with insomnia (98.7% sensitivity and 84.4% specificity; Backhaus, Junghanns, Broocks, Riemann, & Hohagen, 2002). Additionally, the PSQI has been shown to display high correlations with objective measures of sleep latency and duration, such as actigraphy (Boudebesse et al., 2014).

The PSQI does not specifically focus on sleep disorders, and so twins also completed the Insomnia Severity Index (ISI; Bastien, Vallières, & Morin, 2001). The ISI inquired about whether participants displayed any particular symptoms of insomnia in the previous month, as well as the degree of satisfaction with sleep patterns. The measure contained seven items, with scores ranging from 0–28. Higher scores indicate more insomnia symptoms. The ISI had good internal consistency (see Table 1). A cut-off of 10 has been shown to have 86.1% sensitivity and 87.7% specificity in detecting individuals with clinical insomnia (Morin, Belleville, Bélanger, & Ivers, 2011). A subsequent study reported that scores of 9 had 87% sensitivity and 75% specificity in detecting individuals with insomnia (Chung, Kan, & Yeung, 2011). Finally, in individuals receiving treatment for insomnia, the ISI shows significant correlations with sleep diaries and polysomnography (Bastien et al., 2001).

#### Negative affect

Two additional self-report measures assessed negative affect. Traits of depression were measured using the Short Moods and Feelings Questionnaire (SMFQ; Angold, Costello, Messer, & Pickles, 1995), in which participants rated the extent to which 13 statements were true of their typical affective state. Anxiety sensitivity was assessed using the Children’s Anxiety Sensitivity Index (CASI; Silverman, Fleisig, Rabian, & Peterson, 1991), which comprised 18 questions about participants’ fear of the physical symptoms of anxiety.

### Procedure

Questionnaires were mailed out to families who consented to take part in the study. Parents completed the Negative Symptoms subscale of the SPEQ, while twins completed the remaining five SPEQ subscales along with the SMFQ and CASI. All questionnaires were then mailed back to the researchers.

### Data Analyses

#### Data preparation

Any positively skewed measures were log transformed (see Table 1) prior to analyses. The effects of sex and age were regressed out of all measures, as is standard behavioral genetic procedure (McGue & Bouchard, 1984). Analyses were then performed on standardized residual scores. Opposite sex twins were not included in these analyses.

#### Twin analyses

The classical twin design assumes that variance in each trait (‘phenotype’) comprises genetic and environmental components, which are deduced through the differential phenotypic resemblance of MZ twins, who share all their segregating DNA code, and DZ twins, who, on average, share 50% of their segregating DNA code. Multivariate analysis then estimates the degree of genetic and environmental overlap between phenotypes included in the model in a pairwise manner, before partitioning *covariance* between traits into genetic and environmental influences (Purcell, 2008).

First, phenotypic associations between the SPEQ and sleep measures were established using *phenotypic correlations* (*r*_ph_), which were estimated from a multivariate twin model. Twin correlations were then estimated to obtain an indication of the extent of genetic and environmental influences on each phenotype, as well as their covariance with others. Cross-twin correlations involve correlating one twin’s score with their cotwin’s score on the same measure, and were estimated separately by zygosity. Owing to the genetic similarity of MZ twins relative to DZ twins, an MZ correlation higher than the DZ estimate indicates additive genetic (A) influences. Dissimilarities between MZ twins are assumed to be a product of nonshared environment (E), which relates to any environmental factor that differs across twins and creates differences between them. E incorporates measurement error. Hence, if the MZ cross-twin correlation is less than unity, then E is implicated. Shared environmental (C) influences are the opposite of E; these are environmental factors that are common to both twins in a pair, and heighten their similarity. C is indicated when the DZ cross-twin correlation exceeds half the MZ statistic. If, alternatively, the DZ estimate is less than half the MZ estimate, then nonadditive genetic (D) influences, arising from interacting alleles within loci, are suggested.

Cross-trait cross-twin correlations were then estimated to deduce the extent of genetic and environmental influences on the covariance between measures. These correlated one twin’s score on a SPEQ subscale with their cotwin’s score on a sleep measure. These estimates were obtained separately by zygosity, and cannot exceed *r*_ph_ between two traits. The extent of genetic and environmental influences on covariance can be deduced as above for cross-twin correlations. An exception regards how E is derived, which is implicated when the MZ cross-trait cross-twin correlation is less than *r*_ph_. All twin correlations were estimated from a saturated model of the observed data, which constrained variance in each trait to be equal across twins in a pair.

Twin models were then fitted to estimate A, C or D, and E formally. Cholesky decompositions, presented as mathematically equivalent correlated factors solutions (Loehlin, 1996), were fitted to the data. Each phenotype is influenced by three latent variables: A, C (or D), and E. The model then estimates *etiological correlations*, which are essentially an index of the extent to which each etiological influence (A, C, and E) correlates across two traits. They are estimated between 0 and 1. Estimates of 1 indicate that a given etiological influence shows complete overlap across two traits. An estimate of 0 suggests no overlap. These correlations are estimated for additive genetic (*r*_A_), shared environmental (*r*_C_), and nonshared environmental (r_E_) influences. The model then uses estimates of A and *r*_A_ to calculate *bivariate heritability*; the extent of A influences on *r*_ph_ between two phenotypes. Equivalent statistics are calculated for C and E.

**Figure 1 fig1:**
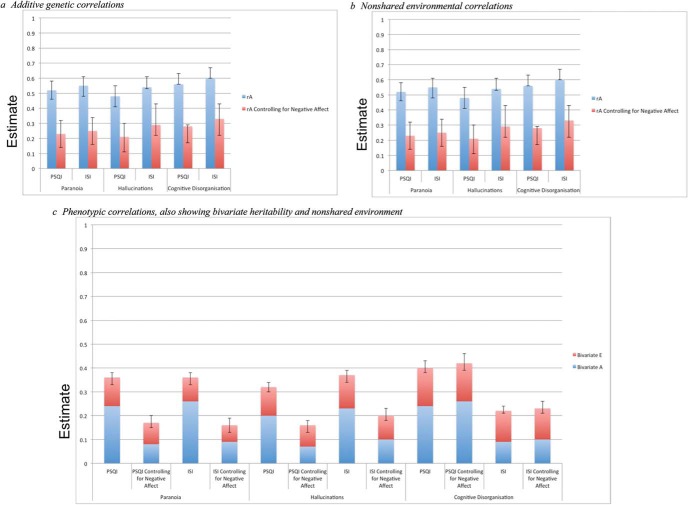
Graphs showing etiological and phenotypic correlations before and after controlling for negative affect: (a) additive genetic correlations, (b) nonshared environmental correlations, (c) phenotypic correlations, also showing bivariate heritability and nonshared environment. PSQI = Pittsburgh Sleep Quality Index; ISI = Insomnia Severity Index. See the online article for the color version of this figure.

Note that the model shown in Figure 1 can readily be extended to include even more phenotypes, as was the case here. Due to model identification constraints, one cannot estimate all four of A, C, D, and E using data collected from twins reared together alone (Purcell, 2008); hence, ACE or ADE models were tested based on the pattern of twin correlations. Each model was fitted twice; once with estimates equated across sexes and with quantitative sex differences, which enabled estimates to differ across sexes. Prior to fitting these models, a saturated model of the observed means, variance, and covariance in the data were fitted. The best fitting model was then selected on the basis of which model fit best relative to the saturated model. For each model, the −2LL fit statistic was obtained. The difference in −2LL between two models is $x^{2}$ distributed, with degrees of freedom equal to the difference in number of estimated parameters. Significant $x^{2}$ results indicate that a given model is a significantly poorer fit relative to the saturated model. This test can be oversensitive to small deteriorations in model fit in large samples, and so two additional indices also assessed model fit: Akaike’s information criteria (AIC) and Bayesian information criteria (BIC). Lower, preferably negative, AIC values suggest better fitting models, while the model with the lower BIC estimate is to be favored. Differences greater than 10 indicate that a particular model is fitting data well (Raftery, 1995).[Fig-anchor fig1]

The best fitting ACE or ADE model was chosen on the basis of the lowest BIC estimate. Within this model, parameters were dropped to test their significance. If dropping parameters did not significantly worsen the fit of the model, then they were removed.

#### Controlling for negative affect

Scores on all measures were subsequently adjusted further for the effects of SMFQ and CASI scores using regression. Scores on both measures were regressed out simultaneously. The best fitting model from the previous analyses was then refitted to these standardized residual scores.

All analyses were conduced in the OpenMx (Boker et al., 2011) and psych (Revelle, 2013) packages of R (R Core Team, 2013).

## Results

### Phenotypic Analyses

Descriptive statistics for the SPEQ subscales, PSQI, and ISI are all shown in Table 1. Table 2 shows *r*_ph_ values between the SPEQ subscales and two sleep measures. Paranoia, Hallucinations, and Cognitive Disorganization displayed moderate, significant correlations with both sleep measures (*r*_ph_ = 0.32–0.42). Negative Symptoms and Anhedonia correlated less strongly (*r*_ph_ = 0.08–0.17), while Grandiosity did not show significant correlations (*r*_ph_ = 0.01–0.04, *ns*).[Table-anchor tbl2]

As the covariance between Grandiosity, Anhedonia, Negative Symptoms, and the two sleep measures was modest, subsequent analyses (twin correlations and twin modeling) were only performed on Paranoia, Hallucinations, Cognitive Disorganization, the PSQI, and the ISI.

### Twin Correlations

Twin correlations are shown in Table 3. For all three SPEQ scales and both sleep measures, MZ cross-twin correlations were higher than DZ estimates, indicating A. No estimate reached 1, however, suggesting that E played a role in all measures. D was implicated for the PSQI and ISI by DZ cross-twin correlations of less than half the MZ statistics, DZ estimates were greater than half the MZ estimates for the three SPEQ scales, indicating C. Cross-trait cross-twin (CTCT) correlations are shown in the lower portion of Table 3; notably, no estimate reached r_ph_, which suggests that covariance between the three SPEQ scales and both sleep measures was partly influenced by E. All estimates were greater for MZ twins than DZ twins, suggesting A on the covariance between traits. The DZ CTCT correlation between Paranoia, Cognitive Disorganization, and both sleep measures were approximately half the MZ estimates, indicating that C did not influence the covariance between these measures. The DZ CTCT correlations between Hallucinations and both sleep measures were larger than half the MZ estimates suggesting that C played a role in the covariance between these measures.[Table-anchor tbl3]

#### Twin Model Fitting

The twin correlations suggested D for the PSQI and ISI, but C for the three SPEQ subscales, hence ACE and ADE models were both tested. All models contained five phenotypes: Paranoia, Hallucinations, Cognitive Disorganization, the PSQI, and the ISI.

The fit statistics for the twin models are shown in Tables 4 and 5. An ACE correlated factors solution had a lower BIC value than an ADE model. Additionally, this model fitted better when estimates were equated across sexes compared to models with quantitative sex limitation. BIC reduced further when C parameters were dropped from the model, hence an AE correlated factors solution was chosen as the best fitting model.[Table-anchor tbl4][Table-anchor tbl5]

The variance components estimated from this model (shown in Table 6), suggest that 41% of the variance in *both* sleep measures was due to A, with the remaining 59% due to E. For the three SPEQ scales modeled (Paranoia, Hallucinations, Cognitive Disorganization), A accounted for 44–51% of the variance, while 49–56% was due to E, as reported elsewhere (Zavos et al., 2014). Etiological correlations are also shown in Table 5. There was moderate A overlap between SPEQ and the PSQI and the ISI. Estimates of *r*_A_ ranged from 0.48–0.60. Estimates of *r*_E_ were modest and significant; they ranged from 0.19–0.28. These overlapping A influences explained most of the covariance between the SPEQ subscales, and PSQI and ISI (see lower portion of Table 6). In total, 60–71% of *r*_ph_ estimates were attributable to A, with the remaining 29–40% of the covariance due to E.[Table-anchor tbl6]

### Controlling for Negative Affect

Scores on the SMFQ correlated significantly with scores on the three SPEQ subscales (*r* = .40–0.58) and both sleep measures (*r* = .66–0.70). CASI scores also correlated significantly with the SPEQ subscales (*r* = .40–0.52) and both sleep measures (*r* = .55–0.56).

Controlling for SMFQ and CASI scores resulted in a significant reduction in the magnitude of the phenotypic and etiological correlations between the SPEQ subscales and PSQI and ISI. Figure 1 presents these estimates against the estimates from the model outlined above. Estimates of *r*_ph_ all reduced, but remained significant, when controlling for negative affect. Between Paranoia and the PSQI and ISI, estimates reduced from 0.36 to 0.16–0.17; for Hallucinations and PSQI and ISI, *r*_ph_ reduced from 0.32–0.37 to 0.16–0.20; and for Cognitive Disorganization, estimates changed from 0.40–0.42 to 0.22–0.23.

Estimates of *r*_A_ also reduced, yet remained significant, after controlling for CASI and SMFQ scores. Between Paranoia and the PSQI and ISI, estimates were 0.23–0.25; for Hallucinations, *r*_A_ with PSQI and ISI was 0.21–0.29; and for Cognitive Disorganization, these estimates were 0.21–0.29. All *r*_E_ estimates reduced when controlling for SMFQ and CASI scores; however, this was only significant between Paranoia and the PSQI (0.14) and Cognitive Disorganization and the PSQI (0.20).

## Discussion

To date, no research has tested whether specific PLEs and sleep disturbances are associated with similar genetic and environmental influences in adolescence. In conducting a twin study of these traits in adolescence, we tested this possibility. We demonstrated, for the first time, that the genetic influences on two positive symptoms (paranoia and hallucinations) and cognitive disorganization displayed a moderate degree of overlap with those on sleep disturbances. Environmental influences on these measures also displayed modest, yet significant, overlap. We also found that the central role of negative affect on this association, widely reported in clinical literature (Freeman et al., 2012), extended to genetic and environmental associations between sleep disturbances and PLEs, which was consistent with our hypothesis.

Prior work has suggested that positive and cognitive PLEs are both linked with sleep disturbances (Lee et al., 2012), and, indeed, our findings tally with this finding. Yet negative symptoms, in contrast with existing work (Lunsford-Avery et al., 2013), did not show such a high degree of overlap with sleep disturbances. A possible explanation for this is that the use of different raters for sleep disturbances, which were assessed via self-report measures, and negative symptoms, which were rated by parents, could have attenuated the phenotypic correlations between these measures. This is unable, however, to explain why self-reported anhedonia, an additional negative psychotic experience, also displayed weak associations with sleep disturbances. It could be that the association between negative symptoms and sleep disturbances becomes more pertinent in adulthood, and that these phenotypes do not co-occur strongly in adolescence, although this hypothesis requires further testing.

As expected, part of the genetic, and some of the environmental, associations between sleep disturbances and PLEs could be explained by the presence of negative affect. This is not surprising, since it is widely reported that negative affect is linked with sleep disturbances (Buysse et al., 2007), and that associations between PLEs and sleep disturbances reduce when controlling for negative affect (Freeman et al., 2012). In extension of these findings, it also appears that negative affect plays a role in accounting for the genetic and associations between sleep disturbances and PLEs. This further emphasizes the important mediating role played by negative affect in the relationship between PLEs and sleep disturbances (Freeman et al., 2010, 2012). Although traits in adolescence are not the same as serious psychiatric disorders in adults, it is noted that the close relationship between negative affect and PLEs seen here as traits in a community sample of adolescence, to some extent, parallels recent molecular genetic results which indicate considerable pleiotropic genetic effects across major depressive disorder and schizophrenia in adults (Cross-Disorder Group of the Psychiatric Genomics Consortium, 2013).

The findings reported here are cross-sectional, and so conclusions regarding the causal relationship between sleep disturbances and PLEs must be taken lightly. Nevertheless, these findings can inform current thinking on the causal links between PLEs and sleep disturbances. One hypothesis is that sleep disturbances, negative affect, and PLEs maintain one another in a cyclical manner (Freeman et al., 2012). Adding to this hypothesis, our findings suggest that shared causes give rise to sleep disturbances and PLEs. Once both have developed, perhaps they then maintain one another in the manner postulated by Freeman et al. (2012), described above. A test of this would be possible with longitudinal twin data; one can assess the direct influence of a trait on another, as well as estimating genetic and environmental contributions to the causal phenotypic relationship between two traits (Luo, Haworth, & Plomin, 2010). Such work would be an informative future direction for this research. Future work hence needs to focus on age groups beyond adolescence in order to investigate the developmental progression of the association between sleep disturbances and PLEs.

Some of the association between PLEs and sleep disturbances was, however, independent of negative affect. In light of this, research endeavors aimed at uncovering the shared molecular genetic and environmental bases of sleep disturbances and PLEs seem warranted. In terms of genetic research, a recent promising avenue regards genetic variants associated with calcium signaling, which have been implicated in clinical schizophrenia and bipolar disorder (Cross-Disorder Group of the Psychiatric Genomics Consortium, 2013; Ripke et al., 2013).

Similarly, a recent genome-wide association study linked genetic loci associated with calcium channels, including CACNA1C, with sleep quality (Parsons et al., 2013), although this finding did not replicate in a subsequent, albeit small-sampled, study (Byrne et al., 2013). The finding that genetic variants associated with schizophrenia were also associated with other psychiatric conditions has led some to speculate that understanding the genetics of other psychiatric conditions will offer inroads into understanding psychotic disorders (Owen, 2012). Our findings, combined with tentatively overlapping molecular genetic findings across psychotic disorders and sleep disturbances, raise the possibility that results from studies of the genetics of sleep disturbances could feed into work on the genetic basis of PLEs.

While clearly a distal goal, uncovering the shared causes of PLEs and sleep disturbances may also prove informative for investigations into their shared neurobiology. As mentioned in the introduction, fluctuations in serotonin levels, and reuptake atypicalities, have been linked with sleep disturbances (e.g., Kostev, Rex, Eith, & Heilmaier, 2014), and, indeed, there are numerous hypotheses that posit that clinical psychotic disorders are similarly linked with serotonin (e.g., Aghajanian & Marek, 2000). As well as linking potential shared causes with specific neurotransmitters, further insight into the shared causes of sleep disturbances and PLEs may be gleaned from objective measures of sleep. A recent study, for instance, reported that atypical sleep spindles were apparent in patients with early onset schizophrenia (Manoach et al., 2014). Given the early onset of these sleep spindle atypicalities in Manoach et al.’s (2014) work, one could question whether such atypicalities emerge even earlier, in individuals presenting with high degrees of PLEs and sleep disturbances, albeit it is necessary to note that such objective abnormalities are not necessarily predictive of poor sleep quality.

We also found a modest significant degree of nonshared environmental overlap between PLEs and sleep disturbances. Nonshared environmental influences are unique to each individual growing up within the same home, and cause differences between them (Purcell, 2008). Separate investigations in relation to PLEs and sleep disturbances have indeed produced some overlapping findings; for example, negative life events have been implicated in both (Cullen, Fisher, Roberts, Pariante, & Laurens, 2014; Barclay, Eley, Rijsdijk, & Gregory, 2011).

In terms of treatments and interventions, could treating sleep disturbances alleviate clinically recognized PLEs? One study showed that cognitive-behavioral therapy for insomnia led to reductions in delusions and hallucinations in a clinical sample (Myers, Startup, & Freeman, 2011). The current study does not speak directly to what or how interventions would work but offers new insight into why sleep disturbances co-occur with positive and cognitive PLEs in adolescence in the community. If these findings generalize to individuals with clinical needs, then they raise the possibility that treatments for sleep disturbances could be effective in relation to PLEs. Uncovering the shared genetic basis of these difficulties would enable one to trace the underlying shared neurobiology. For example, serotonin has been linked with these difficulties (Pritchett et al., 2012; Aghajanian & Marek, 2000). Furthermore, both have been linked with circadian disruptions. For example, disturbed sleep has been implicated in the disruption of transcripts associated with CLOCK genes (Archer et al., 2014), which have tentatively been linked with psychotic disorders (Lamont, Coutu, Cermakian, & Boivin, 2010). It is important to bear in mind that the finding here stem from normal variation in PLEs and sleep disturbance and not from individuals diagnosed with psychotic and sleep disorders, and that negative affect is also important in this association, meaning that any treatment implications need to take this into account.

As with any study, these findings must be taken in light of certain limitations. We focused on two aspects of sleep, sleep quality and insomnia symptoms, since both have been associated with psychotic disorders in research. Other sleep disturbances are also important however; parasomnias, for example, which include regular nightmares and night terrors, are also common in relation to PLEs (Fisher et al., 2014). Owing to our large sample, it was not practical to administer objective assessments of sleep, such as polysomnography. Nevertheless, it will be important for future studies to test whether the findings reported here, using subjective questionnaires, are also apparent for objective measures. Furthermore, we employed cross-sectional data, meaning that a direction for future research should be to focus on the longitudinal links between sleep disturbances and PLEs.

We do not have data regarding which participants have a psychotic disorder for the sample used in this study, nor are we aware of which participants are currently taking medications of any kind. Nonetheless, such individuals are likely to represent a small proportion of the sample given that this is a general population sample, and psychotic disorders typically develop at a later age than adolescence (Laursen et al., 2007; Poulton et al., 2000;). Additionally, the focus of this study was PLEs, rather than psychotic disorders, owing to the increased rate at which these traits co-occur with sleep difficulties (Lee et al., 2012; Lunsford-Avery et al., 2013), and the increased risk of developing psychotic disorders that this co-occurrence represents (Ruhrmann et al., 2010).

It should, nevertheless, be noted that the questionnaire measures used here are more subjective than structured and semistructured assessments in that items may be interpreted differently by individual participants. Hence, studies of objective sleep measures and in-depth assessments of PLEs would be useful to replicate our findings in future. Furthermore, while three SPEQ subscales specify a time-frame for the behaviors and thoughts of interest, the remaining three (Paranoia, Hallucinations, and Cognitive Disorganization) do not, meaning that it is possible that it is possible that the participants are reporting on earlier experiences. Finally, the Hallucinations subscale does not take account of the possibility that certain individuals in the sample may experience hallucinations when falling asleep or due to drug use.

In conclusion, we have shown that sleep disturbances and positive and cognitive PLEs share genetic and environmental influences with one another. As predicted, the association between sleep disturbances and positive and cognitive PLEs is partly tied to the presence of negative affect, but it also remains above and beyond negative affect. An intriguing new avenue for research is to explore what comprises the shared genetic and environmental influences that lead individuals to experience both PLEs and sleep disturbances in adolescence. There are apparent commonalities in terms of proposed neurobiological pathways in the separate literatures on sleep and schizophrenia. The present findings, from a general population sample of adolescents, suggest that a potential shared neurobiology underlying sleep disturbances and PLEs should be explored.

## Figures and Tables

**Table 1 tbl1:** Descriptive Statistics for All Measures

Demographic characteristics		Participating in LEAP (%)		Nonparticipating in LEAP (%)	
Male		45		53	
Monozygotic		35		32	
White		94		91	
		16		12	
Descriptive statistics					
Measure	Cronbach’s α	Possible range of scores	$\bar{x}$ full sample	*SD* full sample	Skew
PSQI	.76	0–21	5.44	2.71	0.92
ISI	.89	0–28	3.76	4.25	1.76
Paranoia	.93	0–72	11.92	10.37	1.50
Hallucinations	.88	0–45	4.51	5.95	2.16
Cognitive Disorganization	.77	0–11	3.98	2.86	0.43
Grandiosity	.86	0–24	5.24	4.37	1.19
Anhedonia	.78	0–50	1.29	1.32	1.14
Negative symptoms	.86	0–30	2.74	3.82	2.37
SMFQ	.88	0.26	3.59	4.42	1.97
CASI	.86	0–36	7.93	5.86	1.13
*Note.* A-levels are advanced qualifications taken in England and Wales at age 17/18. LEAP = Longitudinal Experiences and Perceptions Project; PSQI = Pittsburgh Sleep Quality Index; ISI = Insomnia Severity Index; SMFQ = Short Moods and Feelings Questionnaire; CASI = Children’s Anxiety Sensitivity Index.					

**Table 2 tbl2:** Phenotypic Correlations Between PLEs and Sleep Disturbances

	PSQI		ISI	
	*r*_ph_	95% CI	*r*_ph_	95% CI
Paranoia	0.36	[0.33, 0.38]	0.36	[0.33, 0.38]
Hallucinations	0.32	[0.30, 0.32]	0.37	[0.34, 0.39]
Cognitive Disorganization	0.40	[0.38, 0.43]	0.42	[0.39, 0.44]
Grandiosity	*−0.04*	[*−0.05, 0.01*]	*−0.01*	[*−0.03, 0.04*]
Anhedonia	0.09	[0.02, 0.12]	*0.08*	[*−0.01, 0.11*]
Negative symptoms	0.17	[0.11, 0.23]	0.17	[0.13, 0.21]
*Note.* Italicized estimates are not significant, as indicated by confidence intervals overlapping with 0. The PSQI and ISI correlated .71 (.68/.73). PLEs = Psychotic-like experiences; PSQI = Pittsburgh Sleep Quality Index; ISI = Insomnia Severity Index. *r*_ph_ = phenotypic correlation; 95% CI = 95% confidence intervals.				

**Table 3 tbl3:** Twin Correlations

	MZ		DZ	
Measure	Estimate	95% CI	Estimate	95% CI
Cross-twin correlations				
PSQI	0.43	[0.40, 0.47]	0.16	[0.14, 0.22]
ISI	0.42	[0.37, 0.44]	0.20	[0.17, 0.20]
Paranoia	0.52	[0.52, 0.55]	0.29	[0.29, 0.30]
Hallucinations	0.43	[0.41, 0.48]	0.30	[0.29, 0.35]
Cognitive Disorganization	0.45	[0.42, 0.45]	0.24	[0.20, 0.25]
Cross-trait cross-twin correlations				
Paranoia —PSQI	0.26	[0.22, 0.31]	0.13	[0.12, 0.18]
Paranoia—ISI	0.23	[0.22, 0.28]	0.14	[0.13, 0.19]
Hallucinations—PSQI	0.20	[0.15, 0.22]	0.14	[0.12, 0.20]
Hallucinations—ISI	0.22	[0.17, 0.23]	0.14	[0.12, 0.16]
Cognitive Disorganization— PSQI	0.24	[0.19, 0.25]	0.10	[0.09, 0.11]
Cognitive Disorganization— ISI	0.25	[0.25, 0.25]	0.11	[0.09, 0.13]
*Note.* PSQI = Pittsburgh Sleep Quality Index; ISI = Insomnia Severity Index; MZ = monozygotic twins; DZ = dizygotic twins.				

**Table 4 tbl4:** Twin Model Fit Statistics: Full Models

Model	−2LL	*df*	Parameters	BIC	Δχ^2^	Δ*df*	*p*	AIC
Estimates equated across sexes								
Saturated	74286.91	29,551	130	−165,036.08				
ACE Cor Fac*	74395.64	29,631	50	−165,575.25	108.73	80	.02	−51.27
ADE Cor Fac	74406.63	29,631	50	−165,564.26	119.72	80	<.001	−40.28
Quantitative sex limitation								
Saturated	74093.25	29,421	260	−164,176.92				
ACE Cor Fac	74265.12	29,581	100	−165,300.83	171.87	160	.25	−148.13
ADE Cor Fac	74282.79	29,581	100	−165,283.16	189.54	160	.06	−130.46
*Note.* Cor Fac = correlated factors solution; A = additive genetic influence; D = nonadditive genetic influence; C = shared environmental influence; E = nonshared environmental influence; −2LL = fit statistic; *df* = degrees of freedom; Δχ^2^ = change in −2LL between two models, which is χ^2^ distributed; BIC = Bayesian information criteria; AIC = Akaike’s information criteria.								
* Indicates best-fitting full model; all models fitted to three Specific PLEs Questionnaire subscales (Paranoia, Hallucinations, and Cognitive Disorganization) and the two sleep measures.								

**Table 5 tbl5:** Twin Model Fit Statistics: Nested Models

					Comparison to saturated model				Comparison to ACE decomposition			
Model	−2LL	*df*	Parameters	BIC	Δ$x^{2}$	Δ*df*	p	AIC	Δ$x^{2}$	Δ*df*	*p*	AIC
AE	74422.40	29646	35	−165,669.97	135.50	95	<.001	−54.50	26.76	15	.03	−3.24
CE	74565.67	19646	35	−165,526.70	278.76	95	<.001	88.76	170.03	15	<.001	140.03
E	76014.32	29661	20	−164,199.53	1,727.41	110	<.001	1,507.41	1,618.68	30	<.001	1,558.68
*Note.* All models were nested within the ACE correlated factors solution, with no sex differences. BIC = Bayesian information criteria; AIC = Akaike’s information criteria; A = additive genetic influence; C = shared environmental influence; E = nonshared environmental influence.												

**Table 6 tbl6:** Estimates Derived From the AE Correlated Factors Solutions

	*a*^2^		*e*^2^	
Measure	Estimate	95% CI	Estimate	95% CI
Variance components estimates				
PSQI	0.41	[0.37, 0.45]	0.59	[0.55, 0.63]
ISI	0.41	[0.41, 0.45]	0.59	[0.55, 0.63]
Paranoia	0.51	[0.48, 0.54]	0.49	[0.46, 0.52]
Hallucinations	0.45	[0.41, 0.48]	0.55	[0.52, 0.59]
Cognitive Disorganization	0.44	[0.41, 0.48]	0.56	[0.52, 0.59]
	*r*_A_		*r*_E_	
Etiological correlations				
Paranoia—PSQI	0.52	[0.46, 0.58]	0.22	[0.17, 0.27]
Paranoia—ISI	0.55	[0.48, 0.61]	0.19	[0.14, 0.24]
Hallucinations—PSQI	0.48	[0.41, 0.55]	0.20	[0.16, 0.25]
Hallucinations—ISI	0.54	[0.48, 0.61]	0.24	[0.19, 0.28]
Cognitive Disorganization—PSQI	0.56	[0.56, 0.63]	0.28	[0.24, 0.33]
Cognitive Disorganization—ISI	0.60	[0.60, 0.67]	0.28	[0.23, 0.32]
	Bivariate heritability		Bivariate nonshared environment	
Bivariate heritability and environment				
Paranoia—PSQI	0.67	[0.60, 0.74]	0.33	[0.26, 0.40]
Paranoia—ISI	0.71	[0.64, 0.78]	0.29	[0.22, 0.36]
Hallucinations—PSQI	0.64	[0.56, 0.72]	0.36	[0.28, 0.44]
Hallucinations—ISI	0.63	[0.56, 0.71]	0.37	[0.29, 0.44]
Cognitive Disorganization—PSQI	0.60	[0.53, 0.67]	0.40	[0.40, 0.47]
Cognitive Disorganization—ISI	0.62	[0.55, 0.68]	0.38	[0.32, 0.45]
*Note.* PSQI = Pittsburgh Sleep Quality Inventory; ISI = Insomnia Severity Index; *a*^2^ = additive genetic variance component; *e*^2^ = nonshared environmental variance component; *r*_A_ = additive genetic correlation; *r*_E_ = nonshared environmental correlation; bivariate heritability = proportion of phenotypic correlation due to additive genetic factors; bivariate nonshared environment = proportion of phenotypic correlation due to nonshared environmental factors.
